# Efficacy and Safety of Iodixanol in Computed Coronary Tomographic Angiography and Cardiac Catheterization

**DOI:** 10.3390/jcdd10110449

**Published:** 2023-10-31

**Authors:** Matthew J. Budoff, Hong Seok Lee, Sion K. Roy, Chandana Shekar

**Affiliations:** 1 Department of Cardiology, Lundquist Institute, Harbor-UCLA Medical Center, 1124 W Carson Street, Torrance, CA 90502, USA; sroy@lundquist.org; 2 Mayo Clinic, Cardiology, Phoenix, AZ 85054, USA; lhscd11@gmail.com; 3 Department of Cardiology, College of Medicine, Banner-University of Arizona, 1111 E McDowell Road, Phoenix, AZ 85006, USA; cshekar987@gmail.com

**Keywords:** iodixanol, cardiac CT, coronary CT, CCTA, contrast media, intravenous contrast media, computed tomography, cardiac catheterization, contrast-induced nephropathy

## Abstract

Iodixanol is an iso-osmolar non-ionic dimeric hydrophilic contrast agent with a higher viscosity than the monomeric agents. It is the only Food and Drug Administration (FDA)-approved iso-osmolar agent in the United States, and it is the only contrast agent with an FDA-approved indication for use in cardiac computed tomographic angiography (CCTA), to assist in the diagnostic evaluation of patients with suspected coronary artery disease. In clinical studies, it has been noted to have fewer side effects and similar image quality when compared to low-osmolar contrast media. This can be attributed to the pharmacological properties of iodixanol. These contrast agents are used for coronary computed tomography angiography and cardiac catheterization. In this article, the use, tolerability, and efficacy of iodixanol are reviewed, specifically evaluating the use of CCTA and coronary angiography, including outcome studies, randomized trials, and comparisons to other contrast agents.

## 1. Introduction

In the 1890s, Haschek and Lidenthal used a suspension of chalk and zinnober, a mercury compound, to image the vessels in an amputated hand. Since then, there has been immense research in this area, to develop less toxic and more efficacious contrast agents with more attenuation of X-rays [1]. The ideal intravascular contrast medium is water-soluble, stable, biologically inert, isotonic to plasma, selectively excreted, and available at a reasonable cost [2,3].

Water-soluble contrast agents, based on covalently bound iodine, were first made known by Binz and Rath in the 1920s [4,5]. X-rays are easily attenuated by iodine, as the atomic radius of the covalently bound iodine atoms is 133 picometers, which is in the range of X-ray wavelengths (10 to 10,000 picometers). The covalent bonding to a stable, functional group like benzene also reduces the toxicity of free iodide [6]. Iodinated contrast agents (ICAs) have become quintessential to the practice of radiology and medicine overall. It is approximated that about 75 million doses of ICAs are administered worldwide each year and continue to increase with the use of diagnostic modalities [7].

The ICAs are classified as being ionic or non-ionic (based on the presence or absence of a carboxylate side chain) and monomers or dimers (based on the number of benzene rings). Ionic monomeric agents which dissociate into ions in solution have high osmolality and are hypertonic when compared to plasma. Non-ionic contrast media, which do not dissociate into ions in a solution, have lower osmolality and can be isotonic to plasma [7].

Intravenous contrast administration for computed tomography (CT) scanning is the most common use of iodinated contrast agents. Both catheter angiography and cardiac computed tomographic angiography (CCTA) use intravascular iodinated contrast media for arterial opacification and parenchymal enhancement [6]. This article will review the class of ICAs. Iodixanol (GE Healthcare, Marlborough, MA, USA) is an iso-osmolar non-ionic dimeric hydrophilic contrast agent with a higher viscosity than the monomeric agents [6]. It is the only FDA-approved iso-osmolar agent in the United States, and it is the only ICA with an FDA-approved indication for use in CCTA, to assist in the diagnostic evaluation of patients with suspected coronary artery disease. As such, most studies compare low-osmolar agents to iodixanol, allowing this review to use iodixanol as the reference standard.

## 2. Pharmacological Properties

**Figure 1:**
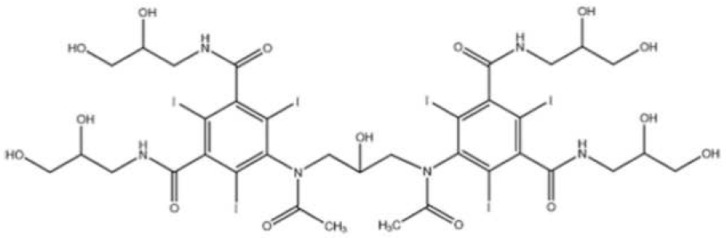
Chemical structure of iodixanol (Visipaque).

Iodixanol is a water-soluble iso-osmolar non-ionic dimeric iodinated contrast agent for intravascular administration. Chemically, it is 5,5′-[(2-hydroxy-1,3-propanediyl) bis (acetylamino)]bis[N,N′-bis(2,3-dihydroxypropyl)-2,4,6-triiodo-1,3-benzenedicarboxamide] (Figure 1), with a molecular weight of 1550.20 and iodine content of 49.1%. It has six iodine atoms per molecule, no carboxyl groups, and nine hydroxyl groups evenly distributed around the molecule [8].

Iodixanol has a similar pharmacokinetic profile as other ICAs. Peak serum concentrations are rapidly achieved, followed by an exponential decrease, with almost no detectable levels in 24 h. Iodixanol is distributed only in the extracellular fluid. Mean distribution half-life (0.43 h), elimination half-life (2.18 h), and apparent volume of distribution (0.275 L/kg) are not dose-dependent [3]. Around 97% of the administered dose is excreted without being metabolized through the urine in 24 h, and the rest through feces in 72 h. This changes to 76% of the dose excreted via urine over five days in patients with stable but severely impaired renal function (creatinine clearance < 20 mL/min), due to prolonged elimination half-life. Non-ionic contrast media does not directly affect the glomerular filtration rate. In healthy individuals, iodixanol acts as a diuretic and produces a dose-dependent increase in urine volume [3]. Iodixanol is available as a solution for injection, at concentrations corresponding to 270 and 320 mg of iodine/mL (mg I/mL). It is mixed with sodium and calcium chloride, which renders it an isotonic solution.

## 3. Image Quality

Higher iodine concentration in the ICA has been attributed to higher mean attenuation in the descending aorta and coronary arteries on CCTA. In a study by Cademitri et al., iodixanol 320 had higher mean attenuation than iohexol (300 mg of iodine/mL) but lower than iohexol (350 mg I/mL), iomeprol (350 mg I/mL), and iomeprol (400 mg I/mL) [9].

Similar results were seen in the study by Honoris et al. [10]. Mean vascular enhancement across all coronary segments was highest in iopamidol (370 mg I/mL), followed by iohexol 350, iodixanol 320, and iodixanol 270, respectively (*p* < 0.002). Image quality, however, graded on a scale of 1–4, by the consensus of two cardiologists, was highest with iso-osmolar iodixanol 320 and 270 (3.27/4.0) and lowest with low-osmolar iopamidol (3.07), (*p* = 0.09) [10]. In the IsoCOR trial (Effect of Iso-osmolar Contrast Medium on Coronary Opacification and Heart Rhythm in Coronary CT Angiography), patients undergoing CCTA were randomized to iso-osmolar iodixanol 270 or low-osmolar iopromide 300. There was no statistical difference in coronary enhancement by the two agents (469 HU ± 167 vs. 447 HU ± 166, respectively (*p* = 0.241)) [11].

The arterial opacification is dependent on the iodine delivery rate, which in turn depends on the rate of contrast injection and concentration of iodine in the contrast medium. To keep a similar level of contrast enhancement (350 HU) during CCTA, iso-osmolar iodixanol 320 needed a higher simulated flow rate (3.90 vs. 3.62 mL/s, *p* = 0.017) and lower iodine delivery rate (1.34 vs. 1.25 g/s, *p* = 0.024) when compared with low-osmolar iopromide 370 in the study by Yang et al. [12]. It has also been suggested that the higher viscosity of iodixanol 320 at 37 °C might also result in a more concentrated intravascular flow pattern, which would lead to similar intra-cardiac iodine concentration as that of iohexol [6]. Appropriate pre-heating of iodixanol is of paramount importance to limit the higher viscosity. When adjusted for iodine concentration in the IsoCOR study, the mean bolus size of iodixanol 270 was larger than that of iopromide 300 (76.8 mL ± 11.6 v/s 69.7 mL ± 10.8, respectively; *p* < 0.001) [13]. Importantly, the voltage used can also affect the attenuation and image quality, with lower kilovolts (<106), demonstrating higher attenuation values than those using 107–120 or higher kilovolts to acquire images. This is true for all contrast types and must be carefully controlled in any comparative study.

Tsai et al. investigated the cardiac enhancement by iohexol 350 and iodixanol 320 on multiple detector computed tomography (MDCT). There was no significant statistical difference between the two agents, though iohexol showed slightly higher enhancement (11.2 HU). However, on the delayed scans, iodixanol had better (7.7 HU) and longer enhancement (*p* < 0.05) in the left ventricle [13]. As dimers have larger molecule sizes than monomers, there is a slower diffusion rate through the capillary pores during the delayed period, leading to longer-lasting attenuation differences between the coronary arteries and myocardial tissue [13]. Less dilution by influx from the extravascular space is expected with decreased osmolarity. This would also be relevant when choosing contrast agents in patients with compromised renal function [14]. Tsai et al. performed a randomized trial of iodixanol 320 and iohexol 350 in 72 patients undergoing MDCT [13]. They showed that enhancement in the right heart, left heart, coronary arteries, and LV myocardium in the arterial phase showed no statistical difference (*p* > 0.05) between the two groups. They concluded that iodixanol 320 can provide vascular enhancement in cardiac MDCT that is similar to iohexol 350 [15]. 

In a prospective multi-center register study with 874 patients by Budoff et al. [16], using cardiac outcomes as the endpoint, the sensitivity of iodixanol-enhanced CCTA was 96.1%, 95.8%, and 94.7% at 1, 6, and 12 months, respectively, with a specificity of 84.5%, 86.6%, and 87.0%, and NPV > 99.0%. Prior trials using iodixanol as the ICA for CCTA have also yielded similar diagnostic accuracy for obstructive coronary artery stenosis, as well as 99% negative predictive value at both the patient and segment levels [17,18]. In a study conducted by Christensen et al., the contrast-to-noise ratio (CNR) was significantly increased for iopamidol 370 versus iodixanol 320 (aortic root, *p* = 0.021; left main, *p* = 0.032; left anterior descending, *p* = 0.033; left circumflex, *p* = 0.039; and right, *p* = 0.009) [18]. Choi et al. performed a double-blinded, randomized, and parallel study on 300 consecutive outpatients who underwent outpatient CCTAs to evaluate CAD. The subjects were assigned to receive either iodixanol 320 or iohexol 350. The image quality of each group was not significantly different from each other. The iodixanol group, however, had a higher CNR (*p* = 0.04) but showed no difference in image noise (*p* = 0.63) and signal-to-noise ratio (SNR) (*p* = 0.07) [15]. In a randomized, double-blinded parallel study with 200 patients undergoing elective cardiac angiography, receiving either iodixanol or iohexol, no statistical difference was noted in the angiographic quality between the two groups (*p* = 0.885) [19]. Similar results were noted in another cardiac angiography study comparing iodixanol and ioxaglate [20].

## 4. Tolerability and Safety

ICAs differ in their tolerability profiles. Iso-osmolarity and lower iodine content have been proven to be safer and more tolerable in humans [10]. The toxicity of poly-iodinated contrast media is a function of the molecule’s physicochemical properties: notably ionicity, higher osmolality and viscosity, and the chemical interaction of reactive iodine anions with biological sites [12].

### 4.1. Pain and Discomfort

Pain and discomfort following contrast administration is not only inconvenient for the patient but can also compromise image quality due to motion artifacts produced by the patient’s body movements during imaging. If the motion artifacts are significant enough to render the study non-evaluable, repeating the study would lead to additional contrast and radiation exposure to the patient and increased cost to the healthcare system. In earlier studies by Klow et al. and Hill et al. in patients undergoing invasive cardiac angiography, the patients receiving iodixanol had a significantly lower incidence of moderate to severe discomfort than those receiving iohexol [19,21]. In a more recent study with patients receiving iodixanol or iopamidol for CCTA, there was no difference in the number of patients having moderate to severe symptoms. However, a subgroup analysis of patients > 55 years of age showed lower rates of moderate to severe symptoms in the iodixanol group (8.5% vs. 24.6%, *p* = 0.01) [22]. In another study, while 27% of patients receiving iodixanol 320 reported pain or warmth, the rate was 13.1% for iodixanol 270, 21.3% for iopamidol, and 16.7% for iohexol (*p* < 0.05) [10]. When compared to a hyperosmolar agent, an iso-osmolar agent has greater vascular stability in arterioles that supply the skeletal muscles and skin over the extremities.

Hence, there is no activation of vascular endothelium and no vasodilation followed by vasoconstriction. The activation of nociceptors in nerves supplying the neurovascular bundles is also less by iso-osmolar agents. This vascular stability and attenuated nociceptor activation may result in less pain and discomfort when iodixanol is used [12]. A study by Murphy et al. showed that diatrizoate, a high-osmolar agent, had higher pain and discomfort rates than low-osmolar iohexol and ioxaglate, thus proving iodine content was not responsible for adverse effects [23]. Hence, it can be hypothesized that the osmolarity of the ICA is the determinant of pain and discomfort after contrast injection and not viscosity or iodine content.

### 4.2. Flushing

Flushing is the most common side effect after intravenous contrast injection. Iodixanol 270 had the lowest incidence of flushing (46%; 95% CI 36–56%) in 513 patients undergoing CCTA when compared to iopamidol (78%; 95% CI 59–87%) and iohexol (72%; 95% CI 55–85) (*p* < 0.001)] [10]. Patients receiving iodixanol had a lower incidence of moderate to severe flushing (3.0% vs. 12.8%, *p* = 0.005) than iopamidol in another study. The difference was more profound in patients over 55 years of age (8.5% for iodixanol vs. 24.6% iopamidol, *p* = 0.01) [22].

Other adverse effects reported, but very infrequently with iodixanol, include chest or leg pain, visual disturbance, dizziness, hypotension, vasovagal reaction, headache, nasal symptoms, taste disturbances, coughing, dyspnea, skin reactions, nausea, vomiting, and shivering [3].

### 4.3. Heart Rate Variability

The patient’s heart rate and its variation play a vital role in cardiac CT imaging due to the limitations in the temporal resolution [24,25,26]. Besides mean heart rate, significant variations in heart rate per minute (due to significant R-R interval variations) also cause misregistration of ECG-gated data, hence causing artifacts in the reconstructed image. These if severe may render the study non-evaluable. Heart rate variability might also potentially increase overall radiation dose, due to longer scan times. Giesler et al. [27] and Hoffmann et al. [28] showed that a multitude of quality measures of CCTA including sensitivity, image quality, and evaluable coronary artery segments was inversely proportional to heart rate. In a CCTA study by Choi et al., median HR change was higher with iohexol when compared to iodixanol (*p* < 0.001) [15,27,28]. While iodixanol showed more HR variation < 3 beats/min (86% v/s 72%, *p* = 0.03), HR variability > 4 beats per min was noted more with iohexol (*p* = 0.003) [15]. In another study, HR variability was lowest for CCTA subjects with iodixanol 320 (2.5 ± 3.0), followed by iodixanol 270 (3.4 ± 5.2), and was highest with iopamidol 370 (4.9 ± 5.8) (*p* < 0.001) [10]. Studies have shown no difference in heart rate variability between iodixanol and iopidamol in patients undergoing CCTA (*p* = 0.56) [22], as well as non-gated chest CT angiography (*p* = 0.72) [29]. No significant difference was seen in the mean deviation of HR in patients undergoing CCTA, receiving iodixanol versus iomeprol (1.4 beats/min v/s 4.4 beats/min, *p* value not significant). However, the latter group showed a greater number of arrhythmic heartbeats during the scan (*p* < 0.001) [30]. Ioxaglate has been shown to induce a greater increase in mean heart rate than iodixanol in patients undergoing cardiac angiography and ventriculography (*p* < 0.05).

Both ioxaglate and iodixanol caused significant QT interval prolongation (*p* < 0.005), but the changes were more marked with ioxaglate [31]. When compared with iomeprol, no significant differences were seen in terms of mean changes in HR during left coronary arteriography (*p* = 0.8), right coronary arteriography (*p* = 0.9), or left ventriculography (*p* = 0.8) by Schmid et al. [32]. The contrast injection during cardiac catheterization could presumably imply more susceptibility to reflex tachycardia, secondary to direct and nondiluted contrast agent injection, than during left ventriculography. Other factors such as the volume of contrast media used, image acquisition time, and route of administration vary during these procedures which might influence the ICA’s effect on cardiac parameters [33]. This could perhaps explain the results from studies where there was no difference in HR variability during left ventriculography between groups who received iodixanol and other ICAs [20,34,35] (Table 1).

## 5. Outcomes

### 5.1. Hemodynamic Changes and Cardiovascular Side Effects

Contrast media have been associated with several cardiovascular adverse effects. Iodixanol had a lesser effect (*p* < 0.01) on left ventricular end diastolic pressure when compared to ioxaglate, in patients with reduced ejection fraction undergoing ventriculography. Large reductions (>20 mm Hg) in diastolic blood pressure occurred in more iodixanol than iohexol recipients during left coronary angiography (13% v/s 3%, *p* = 0.025). Although 62% of patients receiving iodixanol had systolic blood pressure reduction > 20 mm Hg, when compared to 49% receiving iohexol, it was not statistically significant (*p* = 0.07). No differences were noted between the two agents during right coronary angiography or ventriculography [19].

The impact of contrast media with different osmolality on cardiac preload was studied in 90 patients with congestive heart failure (CHF) and chronic kidney disease (CKD), undergoing invasive cardiac angiography with hemodynamic monitoring. The CHF patients were at higher risk when hyperosmolar agents are used, due to the fluid shifts that occur. An increase in extravascular lung water index was seen only when iopromide was used (*p* < 0.001), but global end-diastolic index and central venous pressure significantly increased in both groups (*p* < 0.001, respectively). The overall incidence of acute heart failure was more frequently observed in the iopromide than in the iodixanol group (12 v/s 4 events, *p* = 0.027) [37].

As osmotic pressure was significantly higher in LOCM iopromide than in IOCM iodixanol; the increase in plasma osmolality following LOCM injection could consequently result in an increase in blood volume and cardiac preload.

### 5.2. Major Adverse Cardiovascular Event (MACE)

In a prospective study conducted by Budoff et al. to assess the safety of iodixanol as a contrast agent for CCTA, the rate of MACE (cardiac death, non-fatal myocardial infarction or unstable angina requiring hospitalization) was 5.7% in patients with a positive CCTA (one or more ≥ 50% stenosis) and 0.1% (1/683) in patients with a negative CCTA (99.9% MACE free survival rate) at 12 months follow-up [16].

In a comparison between iodixanol and ioxaglate for angioplasty, the VIP (Visipaque in Percutaneous Transluminal Coronary Angioplasty) trial investigators did not find any significant difference in MACE neither at 2 days (iodixanol, 4.7%; ioxaglate, 3.9%; *p* = 0.45, nor between the 2-day and 1-month follow-ups (*p* = 0.27) in 1141 patients [38]. In the COURT (Contrast Utilization in High-Risk Patients Undergoing PTCA) trial, the in-hospital MACE was 5.4% for iodixanol and 9.5% for ioxaglate (*p* = 0.027). Total events at 30 days in patients who were randomized to iodixanol were also lower (9.1% vs. 13.2%, *p* = 0.07) [39]. In the ICON (Ionic versus non-ionic Contrast to prevent worsening nephropathy after angioplasty in chronic kidney disease patients) trial, however, the use of ioxaglate was associated with lower mortality at l year as compared to iodixanol (2.7% v/s 9.1%, *p* = 0.07) in patients who underwent cardiac catheterization [40]. In a single-center study, patients undergoing percutaneous coronary angioplasty who received iodixanol had more MACE than those who received ioxaglate (4.8% vs. 0.3%, *p* < 0.005). Although the difference was mainly related to the appearance of thrombus during PCI (6% with iodixanol vs. 0.3% with ioxaglate, *p* < 0.0001), use of iodixanol was one of the independent predictors of in-hospital MACE (*p* < 0.001) on multivariate analysis [41]. They concluded that thrombus-related events were more frequent with the iso-osmolar non-ionic dimer iodixanol than with the low-osmolar ionic agent ioxaglate.

In the CONTRAST-AMI (Contrast Media and Nephrotoxicity Following Primary Angioplasty for Acute Myocardial Infarction) trial, no significant difference was found in 1-month MACE in patients who were administered iodixanol and iopromide for the procedure [42]. No significant difference in 1-month major adverse cardiac events was observed (8% in iopromide v/s 6% in iodixanol, *p* = 0.37).

The largest and most robust trial was the Cardiac Catheterization (VICC) trial, in which 1276 patients were randomized, in a double-blind, U.S. multicenter trial comparing the use of iodixanol to iopamidol during percutaneous coronary intervention (PCI) [43]. The primary outcome of the trial was in-hospital major adverse cardiac events (MACE). All events were adjudicated by a clinical events committee blinded to the contrast used. Patients undergoing PCI using iodixanol had 46.7% lower in-hospital MACE (iopamidol 57/630 events vs. 31/646 in the iodixanol group, *p* = 0.003), due to lower rates of non-Q MI (7.5% iopamidol vs. 3.4% with iodixanol, *p* = 0.002). The difference in MI remained significant at 30 days (9.4% vs. 5.3% respectively, *p* = 0.005).

McCullough et al. reviewed 333,533 inpatient coronary or peripheral angioplasties from 357 hospitals [44]. The overall incidence of major adverse renal and cardiac events (MARCE) was 7.41%. There was a 0.69% absolute risk reduction and a 9.32% relative risk reduction in MARCE rate with iodixanol in comparison to LOCM (iohexol, ioversol, iopamidol, ioxaglate, ioxilan, and iopromide). In comparison with LOCM, the use of iodixanol had a 9% decrease in the overall MARCE rate, a 50% decrease in the renal composite endpoint, a 34% decrease in the risk for AMI, a 38% decrease in the risk for angina, and a 21% decrease in the need for repeat stent implantation [44] (Table 2).

Further larger RCTs in patients undergoing CCTA are warranted to avoid the effect of confounding variables that arise with invasive angiography.

### 5.3. All-Cause Mortality

In a study conducted by Wang et al. comparing long-term adverse effects between iodixanol and low-osmolar contrast media, patients who received LOCM had comparable all-cause mortality (n = 6992) with iodixanol, after multivariate regression analysis with adjustments completed for confounding variables. However, in CKD patients, the LOCM group had higher mortality rates than the iodixanol cohort (regression adjusted HR 1.80, 95% CI 0.95 to 3.42) [12]. However, in another study by From et al., there was no difference in all-cause mortality or contrast nephropathy that was seen between iodixanol and iohexol [46].

### 5.4. Contrast-Induced Nephropathy (CIN)

CIN is defined as an acute decline in renal function, expressed as a relative increase in serum creatinine (SCr) concentration of at least 25% or an absolute increase in SCr of 0.5 mg/dL (44.2 mol/L) in the absence of other etiologies [23]. The incidence ranges from 5% in patients with no risk factors to 11–50% in patients with risk factors like renal impairment, congestive heart failure, reduced arterial volume, and use of nephrotoxic medications. Studies have been performed to investigate the nephrotoxic potential of different ICAs [47]. A randomized trial conducted by Chalmers et al. showed that 15% of patients who received iodixanol for angiography had a rise in creatinine of >10% in the week following angiography compared with 31% in the iohexol group (*p* < 0.05) [48]. Another RCT by Aspelin et al. for the NEPHRIC (Nephrotoxicity in High-Risk Patients Study of Iso-Osmolar and Low-Osmolar Non-Ionic Contrast Media) study group, in high-risk patients with diabetes and serum creatinine concentrations of 1.5–3.5 mg/dL, showed that the mean creatinine concentration change from day 0–7 was 0.07 mg/dL in the iodixanol group and 0.24 mg/dL in the iohexol group (*p* = 0.003). The incidence of CIN, defined as a peak rise > 0.5 mg/dL, was decreased from 26% to 3%, (*p* < 0.002) when iodixanol was used. They concluded that iodixanol might have a safer renal profile in high-risk patients than low-osmolar ICAs like iohexol [49].

Similarly, in the Renal Toxicity Evaluation and Comparison Between Visipaque and Hexabrix in Patients with RECOVER (Renal Insufficiency Undergoing Coronary Angiography) study, the nephrotoxicity of iodixanol and ioxaglate was compared as contrast media in patients undergoing coronary angiography. The incidence of CIN was lower with iodixanol (7.9%) than with ioxaglate (17.0%; *p* = 0.021), with an odds ratio (OR) of CIN of 0.415 (95% CI 0.194 to 0.889) for iodixanol. The incidence of CIN was lower with iodixanol in patients with severe renal failure (*p* = 0.023) or associated diabetes (*p* = 0.041) or patients given more volume of contrast media (*p* = 0.038) [50].

Though the ICON study investigators found no statistically significant difference in AKI rates in the iodixanol group (20%) and ioxaglate group (24.3%), it is noted that the study was underpowered [51]. In a meta-analysis with pooled data from 2727 patients, McCullough et al. concluded that the maximum increase in creatinine within three days post-ICA administration was significantly smaller in the iodixanol group when compared with the LOCM group (0.06 mg/dL vs. 0.10 mg/dL, *p* < 0.001). This was more remarkable in CKD patients (0.07 mg/dL vs. 0.16 mg/dL, *p* = 0.004) and CKD combined with DM patients (0.10 mg/dL vs. 0.33 mg/dL, *p* = 0.003). Contrast-induced nephropathy as defined as an increase in creatinine > 0.50 mg/dL within 3 days of contrast use was less in the iodixanol group than in the LOCM group in all patients (1.4% vs. 3.5%, *p* < 0.001), in CKD patients (2.8% vs. 8.4%, *p* = 0.001), and in CKD combined with DM patients (3.5% vs. 15.5%, *p* = 0.003) [44]. Another meta-analysis with 3129 patients showed that intra-arterial iodixanol significantly reduced the risk of contrast-induced acute kidney injury (CI-AKI) when compared with LOCM (RR = 0.68; 95% CI, 0.50–0.92; Z = 2.47; *p* = 0.01) [44]. Nie et al. also found that CIN incidence was significantly lower with iodixanol than with iopromide (5.7% vs. 16.7%; *p* = 0.011) in CKD patients undergoing coronary angiography [52].

No difference in nephrotoxicity was found between iopromide and iodixanol in the DIRECT study [53]. The rate of contrast-induced nephropathy was not statistically different after the intra-arterial administration of iopamidol or iodixanol to high-risk patients, with or without diabetes mellitus, as observed in the CARE (Cardiac Angiography in Renally Impaired Patients) study [54].

When the data of 57,925 patients from the Swedish Coronary Angiography and Angioplasty Registry were compared with the Hospital Discharge Register, clinically significant kidney failure was greatest for patients receiving the iodixanol (1.7%), when compared to ioxaglate (0.8%) (*p* < 0.001). The odds ratio for renal failure in patients with diabetes or prior renal failure was also higher in patients who received iodixanol (*p* < 0.01). The risk associated with iohexol was similar to ioxaglate. The rate of patients needing dialysis was also higher in the iodixanol group (2% v/s 1%, *p* < 0.01) [55]. However, it is likely that the choice of contrast, in a non-randomized sample, could have influenced the outcomes.

Contrast media cause transient endothelium-dependent vasodilation mediated by the release of nitric oxide [56,57]. In the renal blood vessel network, this transient dilation can be followed by sustained vasoconstriction that lasts for several hours, as opposed to peripheral vasoconstriction that lasts for seconds to minutes. In the setting of CKD and DM, there is already a reduction in the renal parenchymal mass and the number of nephrons at baseline. The prolonged vasoconstriction and reduction in blood flow can then impair oxygenation to the medulla, resulting in tubular ischemia. Additionally, the proximal tubular cells are involved in the uptake and release of the ICA in the kidney due to its water solubility. These tubular cells undergo swelling, blebbing, and apoptosis and lead to stasis of the contrast within the kidneys. Also, 3-hydroxy-3-methyl-glutaryl-CoA reductase (HMG Co-A) regulates the production of isoprenoid pyrophosphates, which further play a key role in the proper function of guanosine triphosphate (GTP)-binding protein-mediated endocytosis. The high concentration of contrast within and surrounding renal tubular cells causes direct cellular toxicity. This results in the breakdown of the tubular structures, loss of cell membrane, and removal of material into the urinary tubule (Tamm–Horsfall protein), which leads to more stasis of contrast in the urine, resulting in increased movement of contrast into the tubulointerstitial space, where there is no ready form of clearance. A combination of both ischemic and chemotoxic injury to the proximal tubules triggers tubuloglomerular feedback, which signals the glomerulus to reduce filtration. This results in the rise of the plasma concentration of creatinine, which is seen approximately 24 to 48 h after there has been a significant reduction in filtration [56]. In such high-risk patients, even using the average dose of ICA for routine coronary angiography can cause CI-AKI. There might also be sufficient destruction to some nephrons that recovery might not be possible and fibrosis ensues. In patients with advanced CKD (eGFR < 30 mL·min/1.73 m^2^), CI-AKI can result in a larger reduction in kidney function, accompanied by azotemia, volume overload, and hyperkalemia. The process culminates in progression to the ESRD [56].

Another mechanism that has been proposed for CIN is embolism. As kidneys get 25% of the cardiac output, micro-embolism could account for CI-AKI. The fact that intravenous administration of ICAs has a lower rate of CI-AKI compared to intra-arterial administration, possibly because there is a greater admixture of contrast in the blood pool before it reaches the kidney and there is a lesser chance of athero-embolism, supports this theory [56]. As iodinated contrast is water soluble, it is amenable to prevention strategies that expand intravascular volume, increase glomerular filtration, and promote tubular flow of urine into collecting ducts, ureters, and bladder [56].

### 5.5. Hematological SDE

Contrast agents are also known to cause hemolysis and micro-circulation disturbances. The high viscosity of iodixanol causes an insignificant short-term effect on the microcirculation compared to other agents [57]. In a study conducted by Gerk et al., iodixanol did not cause an increase in free hemoglobin, indicating no hemolysis [58]. In vitro studies conducted by Kerl et al. showed that RBC morphology is less affected by iodixanol when compared with LOCM. This was more significant at higher temperatures at which it is administered, thus not affecting the smooth flow of RBCs in the blood vessels [59].

### 5.6. Neurological SDE

Studies in animals have shown that iodixanol is associated with similar or lesser changes to the blood–brain barrier and neurological function than non-dimeric, non-ionic contrast media, and this is attributed to the hydrophilicity [60].

The primary reason that iodixanol has lower toxicity is that iodexanol is an iso-osmolar, dimeric, non-ionic contrast medium. This lower osmolality and reduced chemotoxicity have generally led to lower osmotic diuresis as compared to those induced by low-osmolar mediums [2,49]. This increased diuresis associated with low-osmolar agents enhances distal sodium delivery, increasing medullary work and potentially inducing hypoxia and volume depletion, with consequent activation of vasoregulatory hormones. This vasoregulatory mechanism difference is likely the cause of increased heart rate variability with low-osmolar agents and potentially the differences in CV outcomes discussed above [42,43,44]. Iodixanol has a lower osmolality and a higher iodine ratio than other non-dimeric contrast media. Furthermore, the presence of hydroxyl groups and the absence of carboxyl groups in iodixanol contribute to reduced toxicity.

## 6. Cost-Effectiveness

The cost of an ICA is important while making a choice, given the contribution to the healthcare cost burden. The cost-effectiveness of iodixanol is not yet clear. A multitude of factors need to be taken into consideration, including the prognosis and number of survival years of patients for whom the agent is used, the healthcare burden of complications an ICA might cause, the healthcare cost for that country or health system, and more. Pricing also varies by institution, depending on the size of the contract, the number and types of products being purchased and involvement in group purchasing, access to cheaper costs with Federal Supply Purchasing, and other factors.

In a study by Iannazzo et al., a base-case analysis showed that intravenous iodixanol was cost-effective compared with LOCM in the Italian clinical setting of a hospital computed tomography radiology practice [61]. Aspelin et al. found that in patients at high risk for CIN, the mean hospitalization cost per patient was €489, €573, and €393 lower after iodixanol than after iohexol using Swedish, German, and French unit prices, respectively [49]. Another European study used Budget Impact Model (BIM) analysis to assess the financial consequences of CIN risk reduction in patients undergoing coronary angiography. Based on the percentage of patients at risk for CIN, they showed that the introduction of iodixanol would bring a 3-year cumulative net percentage saving on the total hospital budgets between 25% and 33% in Germany, Italy, Poland, and Spain [62].

There have been arguments that if this model based on complication risk is applied to studies which did not prove iodixanol to have a superior safety profile when compared to other ICAs, iodixanol would not be considered cost-effective. Based on pricing, Sharma et al. concluded that the price of iodixanol is higher than the price of low-osmolar contrast media in the USA and hence would cost the hospitals more [63]. Keuffel et al. analyzed the cost-effectiveness in the U.S. using BIM for data derived from the Premier Hospital Data [64]. They took into consideration procedural and contrast media prevalence rates, along with MARCE incidence and episode-related cost data. They estimated 513,882 U.S. inpatient angioplasties and 35,610 MARCE cases annually. They applied a previously estimated relative risk reduction in MARCE associated with iodixanol usage (9.3%) and included the higher cost of iodixanol. The annual budget impact was an estimated savings of $30.71 million aggregated across all U.S. hospitals, $6316 per hospital, or $60 per procedure on switching to an iodixanol-only strategy from a LOCM-only strategy [64].

Anaphylaxis: Life-threatening reactions occur in up to 0.2% of individuals depending on the type of contrast used [65]. With the transition from ionic to non-ionic contrast, the risk and rates of anaphylaxis have dropped considerably. Due to very low prevalence, there are no significant differences in rates of anaphylaxis among different non-ionic contrast agents, whether low osmolar or iso-osmolar.

## 7. Conclusions

Iodixanol in coronary angiography either via CT or transcatheter provides good diagnostic accuracy [36,45,66,67], has low heart rate variability, and fewer and less severe side effects with excellent prognostic outcomes, even in patients with prior history of CKD and DM. Overall, for clinical practice, iodixanol has proven prognostic benefits with better sensitivity and better tolerability. Hence, it could potentially serve as a better choice for both invasive and non-invasive coronary angiography.

## Figures and Tables

**Table 1:** Major trials of tolerability.

Authors	Randomized	Number of Patients	Mean Age	Contrast	Endpoints	Results
Lily Honoris et al., 2017 [10]	Yes	N = 513	57 ± 11	Iodixanol Iohexol Iopamidol	Flushing	Iodixanol superior
Xiao et al., 2016 [36]	Yes	N = 2000	52 ± 13.3	Iodixanol 320 Iohexol	Urticaria and nausea	Iodixanol superior
Carlartrand-Lefebvre et al., 2011 [29]	Yes	N = 130	52 ± 16	Iopamido Iodixanol 320	HR	No difference
Jared D. Christensen et al., 2011 [7]	Yes	N = 60	53.5 ± 15.1	Iodixanol 320 Iopamidol 370	HR	No difference
Ryo Nakazato, et al., 2016 [22]	Yes	N = 266	57.2 ± 11.7	Iodixanol Iopamidol	HR Flushing	No difference No difference
Choi et al., 2012 [15]	Yes	N = 300	62 ± 11	Iodixanol 320 Iohexol 350	HR	Iodixanol superior

**Table 2:** Major trials of MACE (major adverse cardiac events).

Study	Number of Pts	Mean Age	Contrast	Endpoint	Results
Qian et al., 2017 [37]	N = 90	62 ± 13	Iodixanol Iopromide	90-Day cardiac event Acute heart failure	Iodixanol superior No difference
Bertrand et al., 2000 [38]	N = 1411	61.6 ± 10.6	Iodixanol Ioxaglate	Acute renal failure	No difference
Davidson et al., 2000 [39]	N = 810	61 ± 12	Iodixanol Ioxaglate	During hospital stay 30-Day cardiac event	Iodixanol superior No difference
Harrison et al., 2003 [43]	N = 1276	-	Iodixanol Iopamidol	In-hospital 30-Day cardiac event	Iodixanol superior No difference
Giustino et al., 2016 [40]	N = 146	71.6 ± 9.9	Iodixanol Ioxaglate	ARF 30-Day events	No difference No difference
Song T2 et al., 2017 [45]	N = 220	54.1 ± 9.8	Iodixanol Iohexanol	ARF	Iodixanol superior

## Data Availability

Not applicable.
